# Effects of Polystyrene Microplastics on Growth and Toxin Production of *Alexandrium pacificum*

**DOI:** 10.3390/toxins13040293

**Published:** 2021-04-20

**Authors:** Chao Liu, Jiangbing Qiu, Zhixuan Tang, Hong Hu, Fanping Meng, Aifeng Li

**Affiliations:** 1 College of Environmental Science and Engineering, Ocean University of China, Qingdao 266100, China; lc8144@stu.ouc.edu.cn (C.L.); asttl@ouc.edu.cn (J.Q.); tangzhixuan@stu.ouc.edu.cn (Z.T.); hhu@ouc.edu.cn (H.H.); mengfanping@ouc.edu.cn (F.M.); 2 Key Laboratory of Marine Environment and Ecology, Ocean University of China, Ministry of Education, Qingdao 266100, China

**Keywords:** microplastics, *Alexandrium pacificum*, chlorophyll *a*, photosynthesis, paralytic shellfish toxins

## Abstract

Microplastics (MP) widely distributed in aquatic environments have adverse effects on aquatic organisms. Currently, the impact of MP on toxigenic red tide microalgae is poorly understood. In this study, the strain of *Alexandrium pacificum* ATHK, typically producing paralytic shellfish toxins (PST), was selected as the target. Effects of 1 and 0.1 μm polystyrene MP with three concentration gradients (5 mg L^−1^, 25 mg L^−1^ and 100 mg L^−1^) on the growth, chlorophyll *a* (Chl *a*), photosynthetic activity (F_v_/F_m_) and PST production of ATHK were explored. Results showed that the high concentration (100 mg L^−1^) of 1 μm and 0.1 μm MP significantly inhibited the growth of ATHK, and the inhibition depended on the size and concentration of MP. Contents of Chl *a* showed an increase with various degrees after MP exposure in all cases. The photosynthesis indicator F_v_/F_m_ of ATHK was significantly inhibited in the first 11 days, then gradually returned to the level of control group at day 13, and finally was gradually inhibited in the 1 μm MP treatments, and promotion or inhibition to some degree also occurred at different periods after exposure to 0.1 μm MP. Overall, both particle sizes of MP at 5 and 25 mg L^−1^ had no significant effect on cell toxin quota, and the high concentration 100 mg L^−1^ significantly promoted the PST biosynthesis on the day 7, 11 and 15. No significant difference occurred in the cell toxin quota and the total toxin content in all treatments at the end of the experiment (day 21). All MP treatments did not change the toxin profiles of ATHK, nor did the relative molar percentage of main PST components. The growth of ATHK, Chl *a* content, F_v_/F_m_ and toxin production were not affected by MP shading. This is the first report on the effects of MP on the PST-producing microalgae, which will improve the understanding of the adverse impact of MP on the growth and toxin production of *A. pacificum*.

## 1. Introduction

Plastic products have been widely used in daily life due to the characteristics of light weight, strong plasticity, insulation and low cost. Millions of tons of plastic products are manufactured worldwide each year, with total production expected to reach around 600 million tons by 2025 [1]. The amount of plastic litter in aquatic and terrestrial environments has increased dramatically over the past few decades [2]. These plastic wastes are fragmented and disintegrated into ubiquitous and long-lasting small microplastics (MP) through physical, chemical and biological processes such as weathering, seawater erosion and biodegradation. Microplastics are commonly defined as plastic particles with the maximal size or aerodynamic diameters less than 5 mm [3]. Typical MP are mainly composed of polyvinyl chloride (PVC), nylons, polyethylene terephthalate (PET), polyethylene (PE), polypropylene (PP) and polystyrene (PS) [4]. A large number of MP debris migrate to the ocean with the surface runoff, and continue to migrate and diffuse through tides, waves and ocean currents. At present, MP are widely distributed in marine environment, from surface seawater to sediments [5,6], and from the Artic to the Antarctic [7,8], as well as in freshwaters [9], such as rivers or lakes.

The ubiquitous MP in aquatic ecosystems potentially threaten aquatic organisms such as phytoplankton, zooplankton, fish and shellfish. Exposure of *Daphnia magna* to MP with particle sizes of 1–5 μm at concentrations of 0.02 and 0.2 mg L^−1^ for 21 days resulted in an increase of mortality [9]. The feeding behavior, growth, development, reproduction and life history of zooplankton can be adversely affected by MP [10], and MP can reduce the assimilation efficiency of shellfish to food, leading to its own energy imbalance [11]. Microalgae, one of the most important primary producers, are also affected by MP in their growth, photosynthesis and chlorophyll content. The adverse effect on microalgae depends on the size, type and concentration of MP [12]. The PVC microplastics (1 μm) had a significant inhibitory effect on the growth of *Skeletonema costatum*, with the maximum growth inhibition rate of 39.7% after 96 h exposure, but the larger size (1 mm) plastic debris had no effects on growth of microalgae [13]. The occurrence of engulfment of MP (1.0 to 2.0 μm) in microalgal cells of both marine *Platymonas helgolandica* var. *tsingtaoensis* and freshwater microalgae *Scenedesmus quadricauda* resulted in a significant reduction in the density of microalgae [14]. Microplastic beads can wrap around the surface of microalgae *Chlamydomonas reinhardtii* and damage their membranes, thus affecting their growth and photosynthetic efficiency [15]. The exposure of polystyrene MP had negative effects on the growth, oxidative stress and cell micro-structure of *Karenia mikimotoi* [16]. Microplastics can cause dysregulation of gene expression in microalgae *Euglena gracilis* involved in cellular processes, genetic information processing, biological systems and metabolism [17]. Microplastic exposure can stimulate the production of extracellular polymers (EPS) by microalgae, which form heterogeneous aggregates with MP particles for sedimentation, thereby reducing MP exposure concentrations [15,18,19,20]. Some species can also use MP as substrates to promote their own growth [19,21].

Species of the genus *Alexandrium* (Dinophyceae) are widely distributed and can form blooms in subarctic, temperate, tropical and subtropical regions [22,23,24]. More than 30 species have been recorded worldwide, some of which produce paralytic shellfish toxins (PST), such as *A. pacificum*, *A. catenella*, *A. minutum*, *A. australiense*, *A. ostenfeldii* [25]. PST consist of a group of more than 50 neurotoxic alkaloids and are produced by several marine dinoflagellates of the genera *Alexandrium*, *Gymnodinium* and *Pyrodinium* as well as some species of freshwater cyanobacteria [26]. The strains of *A. pacificum* and *A. catenella* can produce high levels of a wide range of PST analogues [27,28], such as PST in *A. catenella* strains from Halifax Harbor ranged from 0.17 to 54 fmol cell^−1^ with the major components being C2 and GTX4 [27]. Comparatively, *A. ostenfeldii*, generally produces low concentrations of a more limited PST profile. As typical toxin accumulating organisms, filter-feeding bivalves, such as scallops, mussels and oysters, can accumulate high levels of PST, and transfer them to higher trophic level organisms along food chains. Ben-Gigirey et al. [29] reported that PST analogues were also found in mullets, mackerels, starfish, squids and ascidians after the dinoflagellate *Alexandrium minitum* blooms in Galician coastal waters in Spain. At present, mass mortality of multispecies marine fishes, sea turtle, birds and mammals have been recorded, which were linked to the PST producing dinoflagellate blooms [30,31]. Paralytic shellfish poisoning occurs frequently worldwide due to the consumption of shellfish contaminated with PST, leading to human illnesses and in extreme cases, death [26].

**Figure 1:**
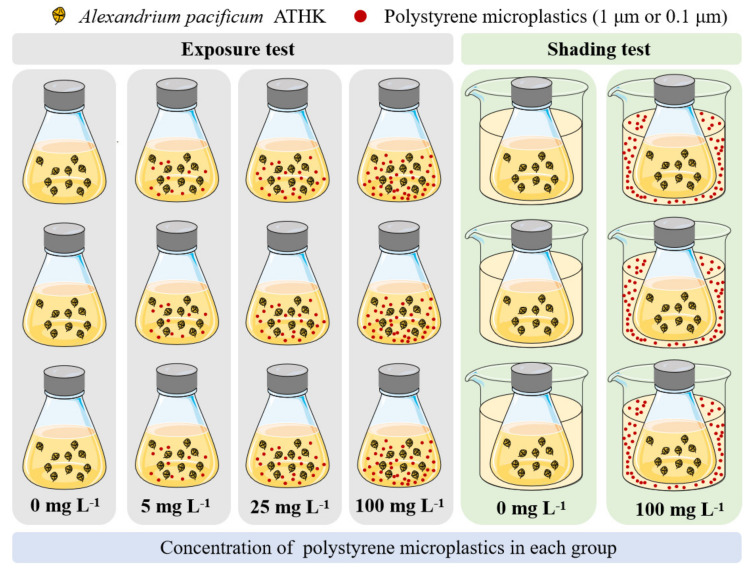
The design of exposure and shading tests of microalgae exposed to microplastics in this study.

At present, the effects of MP on the growth of some freshwater and seawater microalgae have been studied, but the effects on PST-producing microalgae *Alexandrium* species are still unclear. Therefore, the effects of polystyrene MP exposure on *Alexandrium pacificum* growth and PST production were explored in this study. The experiment group of microalgae *A. pacificum* ATHK exposed to different concentrations (5, 25 and 100 mg L^−1^) of polystyrene MP with 0.1 μm and 1 μm and a control group without MP were performed (Figure 1). The cell density, chlorophyll *a*, photosynthetic activity (F_v_/F_m_) and PST of ATHK were also measured. Moreover, the effect of MP shading on the growth and toxin production of microalgae was further evaluated. The data obtained from this study further understand the impact of MP on marine toxin-producing microalgae.

## 2. Results and Discussion

### 2.1. Microalgal Growth

**Figure 2:**
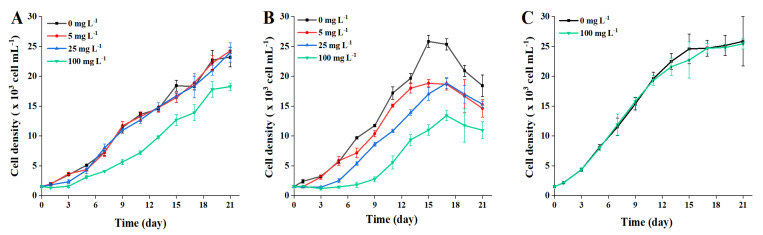
The growth curves of *Alexandrium pacificum* ATHK exposed to different concentrations of 1 μm (**A**) and 0.1 μm (**B**) polystyrene microplastics and shaded by 0.1 μm polystyrene microplastics (**C**).

**Figure 3:**
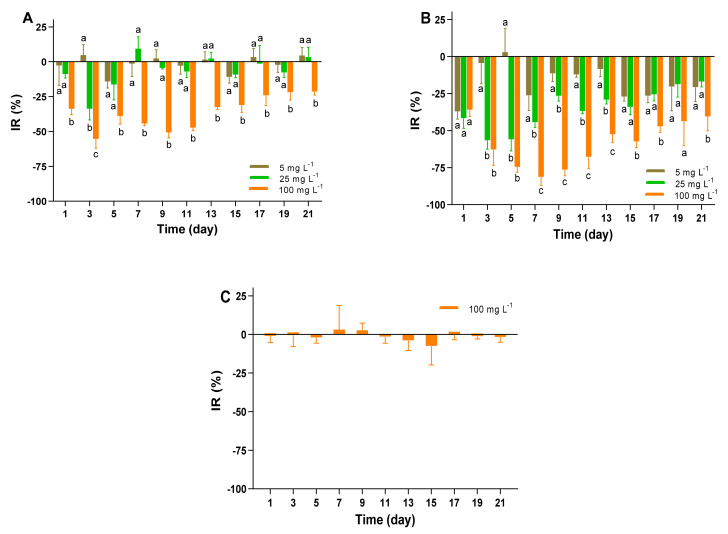
The inhibition rate (IR) of *Alexandrium pacificum* ATHK exposed to different concentrations of 1 μm (**A**) and 0.1 μm (**B**) polystyrene microplastics, and shaded by 0.1 μm polystyrene microplastics (**C**).

Reportedly the microplastics distributed in aquatic ecosystems can interact with microalgae to produce adverse effects, with consequences for growth, photosynthetic activity, chlorophyll content and morphology [12]. As primary producers of aquatic ecosystem, the small-scale disruptions of microalgal populations possibly result in a serious impact on the food webs. Exposure to different concentrations of polystyrene MP, the growth curves of *A. pacificum* ATHK over a 21-days incubation periods are shown in Figure 2. In the exposure treatments of 1 μm MP, the growth of ATHK was significantly inhibited by 21% to 55% during the entire growth cycle in 100 mg L^−1^ treatment group compared to the control group (Figure 3), while it was less affected in other concentrations of MP treatments. No significant difference of the microalgal density was present in the 5 and 25 mg L^−1^ of 1 μm MP treatment groups. The inhibition rate of ATHK exposed to 1 μm MP reached a maximum of 55% in the 100 mg L^−1^ group on the third day of growth and decreased to 21% when they grew on day 21, while their density in the 5 and 25 mg L^−1^ groups was similar as in the control group on day 21. The results indicate that the high concentration of 100 mg L^−1^ of 1 μm MP can significantly inhibit the growth of ATHK, and the effect of MP on the growth of microalgae depends on the exposure concentration, which was consistent with a previous study [32].

However, the growth of ATHK differed markedly when they were exposed to 0.1 μm MP with different concentrations (Figure 2). The inhibition rate (IR) of ATHK increased with the increasing abundance of 0.1 μm MP, indicating that the growth of ATHK was inhibited by MP in a dose-dependent pattern. Exposure to 100 mg L^−1^ of 0.1 μm MP, the density of microalgae barely increased during the first 7 days, and the maximum IR of ATHK was observed on day 7 at 81%. By comparing the adverse effects of polystyrene MP between 0.1 μm and 1 μm particle sizes, it can be easily found that the smaller particles caused higher inhibition impact. A similar difference was also reported in a previous study [32], that showed that the small-size MP (0.05 μm) could significantly inhibit the growth of microalgae *Dunaliella tertiolacta*, while the large-size MP (6 μm) had no significant effect. Generally, the smaller size of MP particles caused the higher toxicity [12,16,32]. Smaller particles may be more easily adsorbed on the surface of microalgal cells to inhibit their growth, such as embedding in microalgal cells, blocking microalgal pores or gas exchanges [13,33]. In addition, different chemical components of MP have different effects on the growth of microalgae [12,20,34,35]. It has been shown that polypropylene MP significantly inhibited the growth of *Chlamydomonas reinhardtii*, but high-density polyethylene MP had no effect on its growth [20]. Zheng et al. [34] also reported the different inhibitory effects of polyvinyl chloride, polystyrene and polyethylene MP on the growth of *Microcystis aeruginosa*. Moreover, MP can also be a substrate to promote the growth of microalgae [21]. The effect of MP on microalgae is also related to the characteristics of microalgal cells, such as size and shape, because the cell wall can hinder particle penetration or affect particle adsorption [33,36]. Overall, the inhibition efficiency of MP on ATHK gradually decreased with the growth of microalgae (Figure 3), which is consistent with the results of previous studies [12,19]. The adverse effect on microalgae caused by MP looks can be repaired by some adaptive strategies of microalgae during a long exposure period. This may be mainly due to the fact that MP can promote the secretion and aggregation of extracellular substances of microalgae, which resulted in the deposition of MP [15,18,20]. In this study, the aggregation and sedimentation of MP particles in the cultures of microalgae was also observed during the later stage (Figure S1), which could reduce the effect of MP on ATHK growth. Shading test of this study showed that MP had no obvious effect on the growth of ATHK (Figure 2), indicating the involvement of other causes for the growth inhibition of microalgae, such as physical damage, osmotic pressure increase and toxic substances release [12,13,19]. Zhang et al. [13] also found that the MP shading effect was not responsible for toxicity of MP on marine microalgae *Skeletonema costatum*, and suggested that the adsorption and aggregation between MP and microalgae may be responsible for the toxicity of MP to marine microalgae.

### 2.2. Chlorophyll a

**Figure 4:**
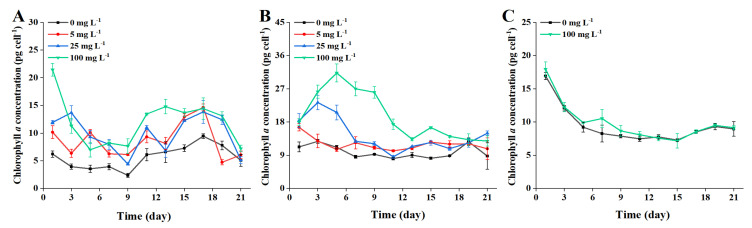
The contents of chlorophyll *a* (fg cell^−1^) in *Alexandrium pacificum* ATHK exposed to different concentrations of 1 μm (**A**) and 0.1 μm (**B**) polystyrene microplastics, and shaded by 0.1 μm polystyrene microplastics (**C**).

Photosynthetic pigments are very important for the photosynthesis and the growth of microalgae. Chlorophyll *a* (Chl *a*), as a light-harvesting pigment complex, is the primary pigment for photosynthesis in microalgal cells, which can reflect the growth and proliferation of microalgae [37,38]. The variation of average content of Chl *a* of ATHK treated with different concentrations of 0.1 and 1 μm MP are shown in Figure 4. Overall, varying degrees of increase for the content of Chl *a* occurred in all MP exposure cases. In the whole growth period, the average contents of Chl *a* in 5, 25 and 100 mg L^−1^ of 1 μm MP treatment groups were 1.7, 1.9 and 2.2 times of that in the control group, respectively (Figure 4). Overall, no remarkable difference in Chl *a* content present among the groups treated with 1 μm MP. Compared with the control group, the exposure of 100 mg L^−1^ of 0.1 μm MP significantly increased the content of Chl *a* in ATKH from day 1 to day 11 of growth (Figure 4), with Chl *a* level 1.6 to 3.2 times higher than that of the control group. The Chl *a* biosynthesis increased by 100 mg L^−1^ of 0.1 μm MP was generally stronger than that by 5 mg L^−1^ and 25 mg L^−1^ of 0.1 μm MP. After the microalgae entered the stable growth period, the effect of MP on Chl *a* biosynthesis gradually decreased. In short, the content of Chl *a* increased after exposure to different concentrations of 0.1 μm and 1 μm MP compared to the control group. Several studies have also observed that Chl *a* content of microalgae *Cladocopium goreaui* and *Dunaliella salina* increased significantly after exposure to MP [36,39]. The increase in chlorophyll a content may be associated with cell growth impairment [39]. However, some studies have showed that MP exposure caused a decrease in Chl *a* content of microalgae. Such as, Chl *a* content of *Skeletonema costatum* was reduced by 20% after 96 h exposure to 50 mg L^−1^ polyvinyl chloride MP [13]; polystyrene MP of 50 mg L^−1^, 100 mg L^−1^, 1000 mg L^−1^ significantly reduced growth and Chl *a* content of *Chlorella vulgaris* [40].

In this study, the shading test of MP had no effect on Chl *a* content (Figure 4). The increase in Chl *a* might be an active response to the microalgal cell damage caused by MP, which try to absorb as much light energy as possible by improving chlorophyll content [41]. In addition to Chl *a*, a variety of photosynthetic pigments such as Chl *b* and carotenoids also exist on chloroplast membrane, all of which are involved in absorbing light energy for photosynthesis [17,42]. The contents of these photosynthetic pigments in microalgae may be affected by MP [43], such as Chl *b* and carotenoids were more sensitive to polystyrene MP than Chl *a* in freshwater microalgae *Euglena gracilis* [17]. Moreover, the strain of *A. pacificum* ATHK is a PST-producing dinoflagellate, and the increase in Chl *a* content may also be related to PST biosynthesis in microalgal cells. Previous studies have shown that the production of domoic acid was closely related to chlorophyll content of *Pseudo-nitzschia multiseries* and DA production decreased or stopped when cellular Chl *a* concentration decreased to a critical level [44].

### 2.3. F_v_/F_m_

**Figure 5:**
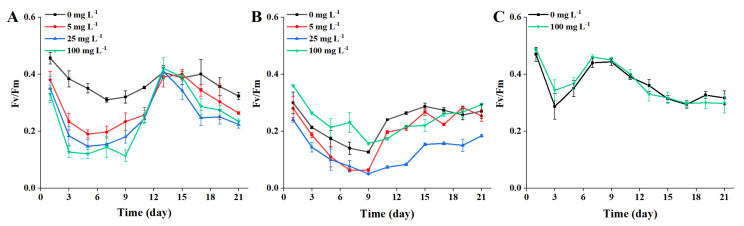
The photosynthesis indicator F_v_/F_m_ of *Alexandrium pacificum* ATHK exposed to different concentrations of 1 μm (**A**) and 0.1 μm (**B**) polystyrene microplastics, and shaded by 0.1 μm polystyrene microplastics (**C**).

An indicator of PS II activity, F_v_/F_m_, is the largest photochemical quantum yield of PS II reaction centrals when all PS II reaction centers are in an open state [45]. The ratio value of F_v_/F_m_ was relatively stable under normal conditions and was not vulnerable to growth conditions, however this value would decrease if the plant was inhibited by light [35,46]. Previous studies have revealed that MP had influences on microalgal photosynthesis [12,15,19]. In this study, the effects of different concentrations of 1 μm and 0.1 μm MP on F_v_/F_m_ of ATHK are shown in Figure 5. Compared with the control group, F_v_/F_m_ was significantly inhibited by 1 μm MP exposure at all concentrations during the first 11 days (Figure 5), and the inhibition rates of F_v_/F_m_ by 5, 25 and 100 mg L^−1^ MP were 17~46%, 23~58%, and 28~67%, respectively. On day 11 to 13, the inhibitory effects of 1 μm MP exposure was attenuated, with almost no significant difference from the control group (*p* > 0.05), then was gradually intensified again on day 15 to 21. Variation of the inhibitory effect of 1 μm MP on F_v_/F_m_ with the growth of ATHK was consistent with the results reported in a previous study [13].

In the 0.1 μm MP-treated groups, compared with the control group, the F_v_/F_m_ responses were different during the first 9 days (Figure 5), with 100 mg L^−1^ MP promoting an increase of 20% to 64% in the F_v_/F_m_ response, but 5 mg L^−1^ and 25 mg L^−1^ MP inhibiting the F_v_/F_m_ response by 6.7% to 55% and 20% to 61%, respectively. In the 100 mg L^−1^ of 0.1 μm MP treatment group, F_v_/F_m_ was first promoted, then inhibited, and finally returned to the level of the control group. The increase of F_v_/F_m_ may be related to the observation of 100 mg L^−1^ of 0.1 μm MP floating on the surface during the first few days of the experiment, which reduced the interaction between MP and microalgae. Although F_v_/F_m_ was not inhibited, microalgal growth was suppressed, which may be related to the negative impact of PS I reaction center or chlorophyll-protein complexes [39]. In the 5 mg L^−1^ of 0.1 μm MP treatment group, F_v_/F_m_ also finally returned to the control level, which was closely related to the reduction of MP effects on microalgae by extracellular substances and MP aggregation and sedimentation. However, in the 25 mg L^−1^ MP (0.1 μm) treatment group, F_v_/F_m_ was still significantly inhibited at the end of stable growth. Overall, compared with other groups, 25 mg L^−1^ of 0.1 μm MP group had the worst effect on the F_v_/F_m_ of ATHK. The inhibition of F_v_/F_m_ shows that MP may cause damage to the PS II reaction centers of ATHK, interrupting the photosynthetic electron transfer process in PS II, leading to a decrease in F_v_/F_m_ and an impact on photosynthesis. Photosynthesis of microalgae is complex and contains PS II and I reaction centers, with multiple chlorophyll and light-harvesting complexes involved in the process. No direct link present between the changes in Chl *a* content and the F_v_/F_m_ ratio. Su et al. [39] also reported that MP can depress photosynthesis of microalgae by reducing the formation of light-harvesting complexes without reducing chlorophyll content and photochemical efficiency.

### 2.4. PST Production

**Figure 6:**
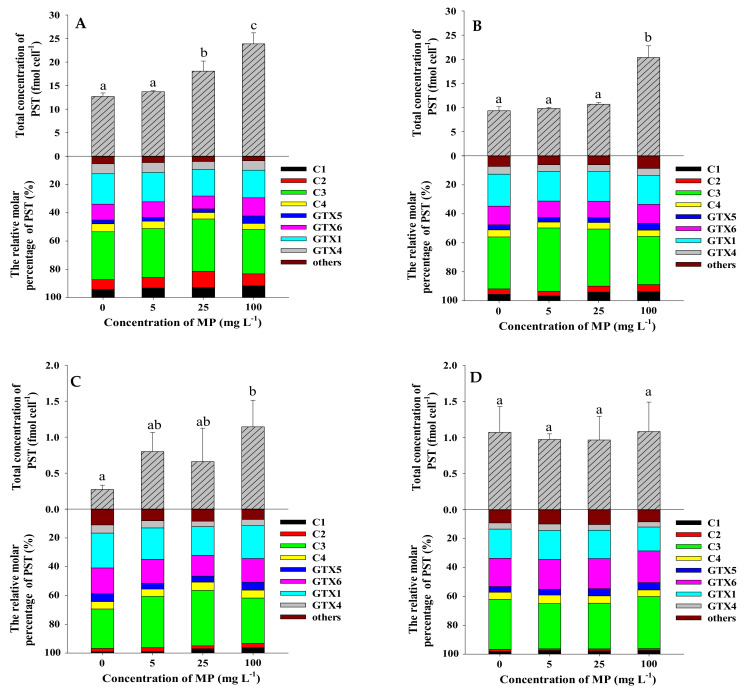
The total concentration (fmol cell^−1^) and relative molar percentage (%) of PST in *Alexandrium pacificum* ATHK exposed to 1 µm polystyrene microplastics on the 7th day (**A**), 11th day (**B**), 15th day (**C**) and 21st day (**D**) of growth. Different letters indicate significantly different values at *p* < 0.05.

**Figure 7:**
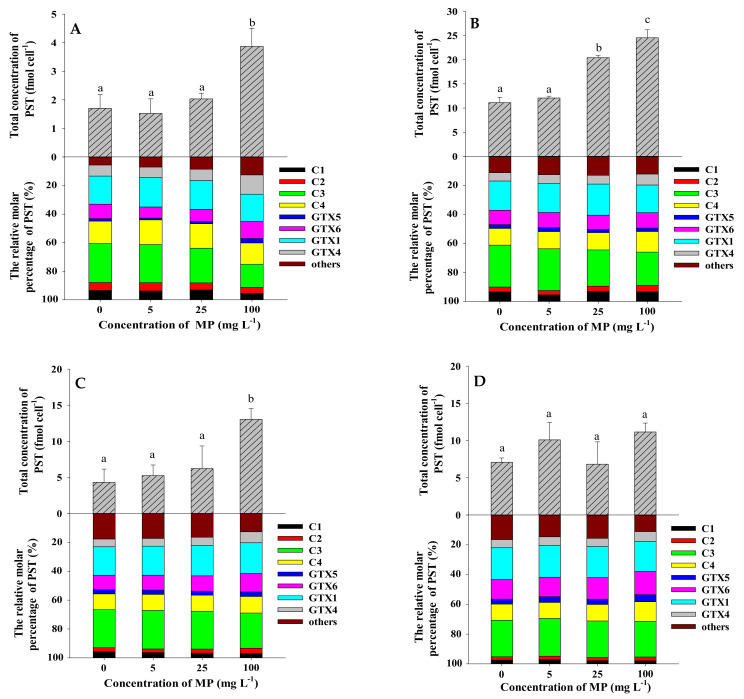
The total concentration (fmol cell^−1^) and relative molar percentage (%) of PST in *Alexandrium pacificum* ATHK exposed to 0.1 µm polystyrene microplastics on the 7th day (**A**), 11th day (**B**), 15th day (**C**) and 21st day (**D**) of growth. Different letters indicate significantly different values at *p* < 0.05.

The strain of *A. pacificum* ATHK is a PST-producing dinoflagellate, mainly producing C1/2, C3/4, GTX1/4, GTX5 and GTX6 and trace amounts of GTX2/3, dcSTX, NEO, M1, M5, M3, M7 and M9, which is consistent with our previous studies [27,47]. Such as, the *N*-sulfocarbamoyl C1-4 toxins accounted for the majority of the toxin (52.2 mol%), followed by GTX1/4 (28.6 mol%) and GTX6 (11.2 mol%), and lower levels of GTX5 and other analogues in ATHK on the 7th day (Figure 6). To determine whether MP can promote the PST production in ATHK, the cell toxin quota (fmol cell^−1^), the total toxin content (nmol L^−1^) and relative molar percentage (%) of PST in response to 0.1 μm and 1 μm polystyrene MP exposure at different concentrations on the 7th, 11th, 15th and 21st day of growth were measured (Figure 6 and Figure 7). Due to the limited space in the light incubator used in the laboratory, the three groups of experiments in this study were conducted in three separate batches, thus resulting in differences in the toxin content of ATHK in each group, but this did not affect the comparison between treatments with different concentrations of MP within the same group.

Compared to the control group, MP at 5 mg L^−1^ did not significantly change the cell toxin quota (*p* > 0.05), regardless of MP particle size. This indicates that the concentration of 5 mg L^−1^ polystyrene MP with size 0.1 μm and 1 μm do not affect the toxin production of individual ATHK cell. Under 25 mg L^−1^ MP exposure, the cell PST quotas collected at all-time points were not significantly different from the control group, except for an increase of 42% and 84% in the 1 μm group on day 7 and the 0.1 μm group on day 11, respectively. When exposed to 100 mg L^−1^ of 1 μm and 0.1 μm MP, the cell toxin quotas were significantly increased by 88% to 319% on days 7, 11 and 15 compared to the control group, while it was not significantly different on day 21. The results showed that although the exposure of 100 mg L^−1^ of polystyrene MP inhibited the growth of ATHK, it promoted the biosynthesis of PST in individual cell of microalgae. At the end of the experiment (day 21), there were no significant difference in the cell toxin quotas treated with different concentrations and particle sizes of MP, which could be caused by the settling of MP at the later stage. Although the cultures were manually agitated daily, the occurrence of MP settling could be seen after 30–60 min of shaking at the later stage. Based on these results, it can be found that only high concentration (100 mg L^−1^) polystyrene MP can significantly promote the production of PST in individual ATHK cell. In terms of total PST content in cultures of *A. pacificum* ATHK (Table 1), 1 μm MP treatments with all concentrations had no significant effect during all periods, except for an increase of 55% in the 25 mg L^−1^ group on day 7 compared to the control group. In the 0.1 μm MP treatments, the total PST contents were not significantly different from the control group in the 5 mg L^−1^ and 25 mg L^−1^ MP groups during all periods, and were reduced by 51% and 29% in the 100 mg L^−1^ MP group on day 7 and 11, respectively, with no effect in all subsequent periods. In summary, the total PST contents and cell quotas of PST in ATHK were barely affected by 5 mg L^−1^ and 25 mg L^−1^ MP, and were more affected by 100 mg L^−1^ MP. All MP treatments did not change the toxin profiles of ATHK, nor did the relative molar percentage of main PST components. Zheng et al. [10] reported that the content of microcystin in *Microcystis aeruginosa* had a noticeable increase after exposure to three types of MP for 96 h, as well as the increase of microcystin content with increasing polystyrene MP concentration (0–100 mg L^−1^). At present, the effects of MP on the toxin production by toxigenic microalgae were less studied and poorly understood.

**Figure 8:**
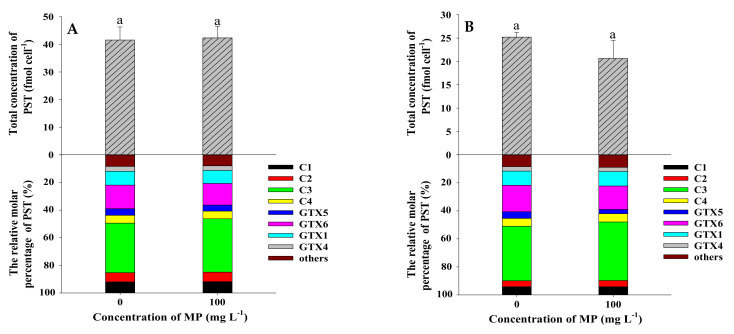

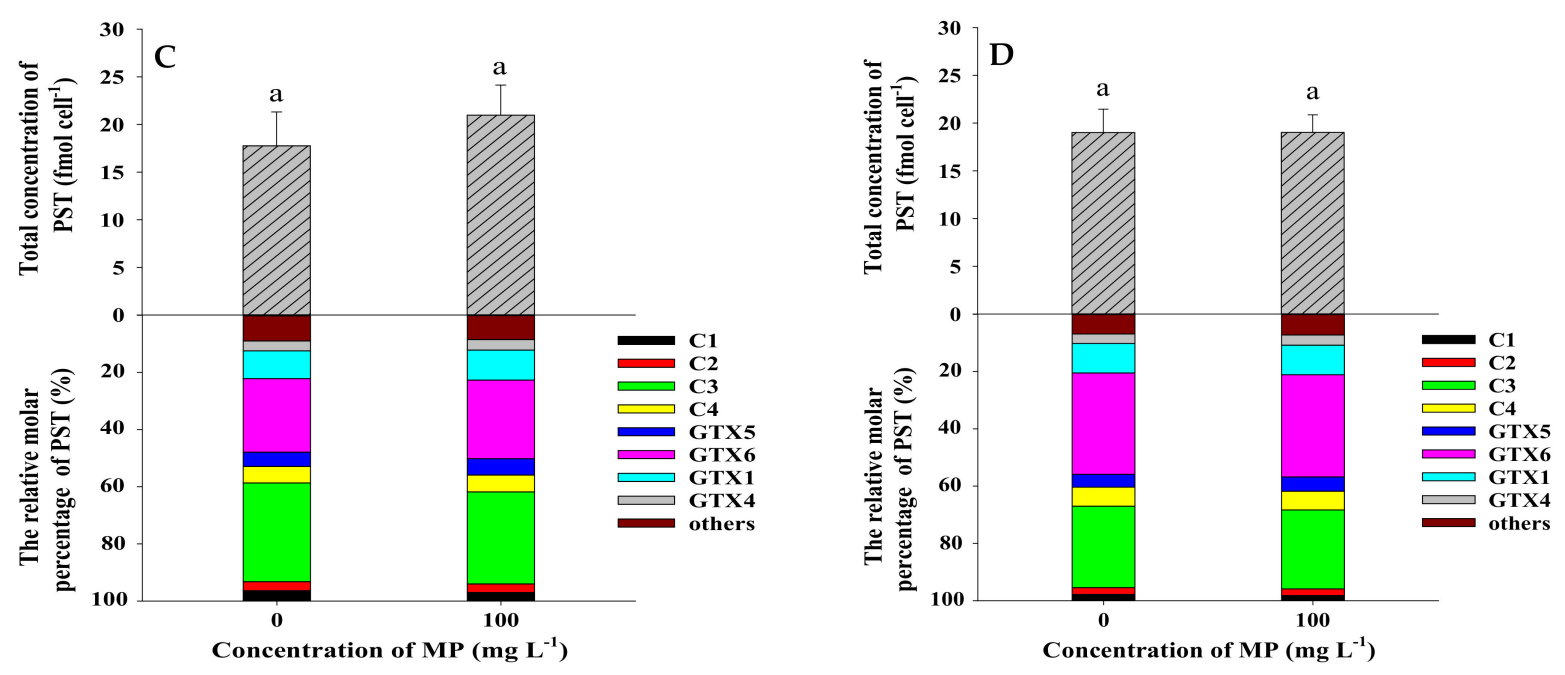
The total concentration (fmol cell^−1^) and relative molar percentage (%) of PST in *Alexandrium pacificum* ATHK Scheme 0. µm polystyrene microplastics on the 7th day (**A**), 11th day (**B**), 15th day (**C**) and 21st day (**D**) of growth. Different letters indicate significantly different values at *p* < 0.05.

The content and composition of PST in *Alexandrium* species were influenced by various factors such as culture conditions (light, temperature, salinity, nutrient levels) and various environmental contaminants [48,49,50,51,52,53]. The PST content in the *A. catenella* strain ACT03 was stable at irradiance ranging from 10 to 70 μmol photons m^−2^ s^−1^, then slightly increased at 130 to 260 μmol photons m^−2^ s^−1^ [48]. It was suggested that light availability could affect the toxin biosynthesis of dinoflagellate *A. catenella* [54]. In this study, shading test showed that MP shading had no significant effect on the average toxin content in each cell of ATHK and the total toxin production (Figure 8 and Table 1), which indicates that MP do not affect the biosynthesis of PST by shading reduced irradiance. The effect of MP on toxin biosynthesis may be due to other factors and needs to be further explored.

## 3. Conclusions

In this study, we investigated the effect of polystyrene MP with different concentrations on the growth, Chl *a* content, F_v_/F_m_ and PST production of *A. pacificum* ATHK. The high concentration (100 mg L^−1^) of MP significantly inhibited the growth of ATHK, with a maximum inhibition rate of 81% at day 7 exposed to 0.1 μm MP. Either the high concentration or the small particle size of MP caused the highest inhibition rate. The biosynthesis of Chl *a* was increased after exposure to MP regardless of its particle size and concentration. In the 1 μm MP treatments with different concentrations, F_v_/F_m_ of microalgae was first significantly inhibited, then gradually returned to the level of control group, and finally was gradually inhibited. In the 0.1 μm MP-treated groups, F_v_/F_m_ of microalgae was first promoted, then inhibited, and finally returned to the level of the control group in the 100 mg L^−1^ treatment, and were differentially inhibited in both 5 and 25 mg L^−1^ exposure groups. The total PST content and cell quota of PST in ATHK were barely affected by 5 and 25 mg L^−1^ MP, and were more affected by the 100 mg L^−1^ MP. The effect of MP on toxin production was temporary, and finally returned to normal level. The growth and toxin production of ATHK were not affected by MP shading. The finding of this work will be helpful for further evaluation of the effects of MP on the growth and toxin production of toxigenic marine microalgae.

## 4. Material and Methods

### 4.1. Chemicals

Polystyrene microplastics (sphere, 25 mg mL^−1^, dissolved in deionized water) with particle size of 0.1 μm and 1 μm were purchased from BaseLine ChromTech Research Centre (Tianjin, China). Certified reference materials of C1/2, GTX1/4, GTX2/3, GTX5, GTX6, STX, NEO, dcGTX2/3, dcSTX and dcNEO were obtained from National Research Council Canada (Halifax, NC, Canada). Acetonitrile was purchased from Merck KGaA (Darmstadt, Germany). Formic acid, ammonium formate and acetic acid were purchased from Fisher Scientific (Fair Lawn, NJ, USA). All reagents and solvents were analytical or HPLC grade. Deionized water (18.2 MΩ cm quality or better) was obtained from a MilliQ water purification system (Millipore SAS, Molsheim, France).

### 4.2. Organisms

The strain of *A. pacificum* (code as ATHK) used in this study was isolated from the coast of Hong Kong, China, and cultured in the laboratory over ten years. The strain can produce a variety of PST, including C1/2, C3/4, GTX1/4, GTX5 and GTX6 [47]. The microalgae were cultivated in sterile seawater filtered with 0.45 μm membrane (Xingya Ltd., Shanghai, China). The culture was enriched by f/2 medium without silicate [55] at 16 °C under 100 μmol m^−2^ s^−1^ photon flux density with a cycle 12 h light:12 h dark. The microalgal cells of *A. pacificum* were counted and collected at log phase growth stage for MP exposure and shading experiments.

### 4.3. Exposure Test

The design of the exposure test is shown in Figure 1. The strain ATHK was cultured in a 2-L conical flask with 1 L of microalgal cultures at an initial density of approximately 1500 cells mL^−1^. Then particle size of 1 μm polystyrene MP were separately added to the microalgal medium, and the final concentrations of polystyrene MP was 0 (control group), 5, 25 and 100 mg L^−1^ in four exposure groups. The low concentrations (5 mg L^−1^) of MP were mainly used to evaluate the effect of environmental concentration of MP on ATHK, and the exposure to high concentrations (25 mg L^−1^, 100 mg L^−1^) was focused on evaluating the potential hazard of MP to ATHK. Each group was in triplicate. The test conical flasks were randomly placed in illuminated incubator for 21 days under the condition in accordance with pre-cultured condition and were manually shaken gently three times a day for 10 s each time to prevent the sedimentation of microalgae and MP. Three milliliters of microalgal cultures were taken out for the measurement of cell density, Chl *a* and F_v_/F_m_ every 48 h. After 5, 11, 15 and 21 days, 50 mL of microalgal cultures were taken for PST analysis.

There was a significant difference in the growth of ATHK between the experimental group and the control group after exposure to 1 μm MP, so another batch of *A. pacificum* ATHK was also used to carry out 0.1 μm polystyrene MP exposure experiment. The experimental design, conditions and procedure were the same as above.

### 4.4. Shading Test

The design of the shading experiment is shown in Figure 1. The strain ATHK was cultured in a 500 mL conical flask with 400 mL of microalgal cultures at an initial density of approximately 1500 cells mL^−1^. A 1-L beaker was added with 250 mL of f/2 medium and 0.1 μm polystyrene MP, and the final concentration of MP was 100 mg L^−1^ in the beaker. The control group did not add MP and contained only 250 mL of f/2 medium. The conical flask was then placed in a beaker, and each group was in triplicate. The experimental conditions and procedure were the same as above.

### 4.5. Determination of Microalgal Density, Chlorophyll a and F_v_/F_m_

One milliliter of microalgae was taken and filtered through a 50-μm sieve into a centrifuge tube, and the cell density was determined by an Accuri C6 Plus flow cytometer (BD Biosciences, NJ, USA). The growth curve of 21 days was drawn, and growth inhibition ratio (IR) was calculated as: *IR*(%) = (1−*T*/*C*) × (−100%), where *T* and *C* were cell density in experimental group and control group respectively. Chlorophyll *a* content and the maximum quantum yield (F_v_/F_m_) were determined by the pulse amplitude modulated fluorometer device PHYTO-PAM^®^ Fluorometer Analyser (Walz, Effeltrich, Germany) according to Pang et al. [56]. Microalgal culture medium (1.5 mL) was collected and incubated in the dark for 15 min before fluorescence measurements. The initial fluorescence parameters (F_0_), as the basal fluorescence of photosystem II (PS II), was measured with modulated light of 1 μmol photons m^−2^ s^−1^. The maximum fluorescence (F_m_) were measured after a saturating flash of 1064 μmol photons m^−2^ s^−1^. The variable fluorescence (F_v_) was determined by the difference between F_0_ and F_m_. Based on the values of these fluorescence parameters, the maximum quantum yield of PS II (F_v_/F_m_) was obtained. The concentration of the Chl *a* was determined using an irradiance of 32 μmol photons m^−2^ s^−1^.

### 4.6. Toxin Extraction

The extraction of PST from microalgal cells was according to our previous study [43]. In brief, 50 mL of microalgal cultures were centrifuged at 6000× *g* for 10 min to remove the supernatant, then pellets of microalgae were suspended in 2 mL of 0.1 mol L^−1^ acetic acid and vortex mixed for 1 min. Samples were frozen and thawed in liquid nitrogen for three times and sonicated for 4 min using a sonication probe (KS-750F, Kesheng Ultrasonic Equipment Ltd., Ningbo, China). The toxin extract was centrifuged at 6000× *g* for 10 min at 4 °C and the supernatant was filtered through a 0.22 µm membrane into a vial for LC-MS/MS analysis.

### 4.7. LC-MS/MS Analysis

Samples were analyzed using a Thermo U3000 HPLC (Thermo Fisher Scientific, Bremen, Germany) coupled to an AB Sciex Qtrap 4500 mass spectrometer (AB Sciex Pte. Ltd., Singapore). Both LC and MS source conditions were as described by our previous study [57]. In brief, a TSK-gel Amide-80 HILIC column (250 × 2 mm inner diameter, 5 μm, Tosoh Bioscience LLC) was used to separate PST using a binary mobile phase of solvent A (water containing 2.0 mmol L^−1^ formic acid and 50 mmol L^−1^ ammonium formate) and solvent B (100% acetonitrile).

### 4.8. Statistical Analysis

All data were expressed as means ± standard deviations (*n* = 3). One-way analysis of variance (ANOVA) followed by Least Significant Difference (LSD) test was employed to identify significant differences between different groups (α = 0.05) using the SPSS statistical package version 25. The letters indicate the results of analysis of variance and different letters indicate significantly different values at *p* < 0.05. All figures were drawn by the software SigmaPlot 14.0.

## Figures and Tables

**Table 1:** Total PST content in cultures of *Alexandrium pacificum* ATHK (nmol L^−1^).

MP Concentration (mg L^−1^)			7 d	11 d	15 d	21 d
Exposure test	1 μm	0	92 ± 3.0 ^a^	127 ± 15 ^a^	5.0 ± 1.3 ^a^	25 ± 12 ^a^
		5	99 ± 11 ^a^	130 ± 14 ^a^	13 ± 5.2 ^a^	24 ± 1.7 ^a^
		25	143 ± 10 ^b^	135 ± 3.3 ^a^	16 ± 8.6 ^a^	23 ± 10 ^a^
		100	97 ± 9.5 ^a^	146 ± 18 ^a^	15 ± 7.2 ^a^	20 ± 9.4 ^a^
	0.1 μm	0	17 ± 6.2 ^a^	171 ± 6.6 ^a^	105 ± 58 ^a^	125 ± 27 ^a^
		5	10 ± 3.0 ^ab^	168 ± 6.0 ^a^	92 ± 28 ^a^	137 ± 24 ^a^
		25	11 ± 1.7 ^ab^	198 ± 2.9 ^a^	100 ± 66 ^a^	100 ± 55 ^a^
		100	8.3 ± 2.2 ^b^	122 ± 35 ^b^	134 ± 23 ^a^	116 ± 23 ^a^
Shading test	0.1 μm	0	475 ± 39 ^a^	493 ± 23 ^a^	428 ± 47 ^a^	482 ± 26 ^a^
		100	497 ± 52 ^a^	400 ± 84 ^a^	469 ± 35 ^a^	482 ± 32 ^a^

Note: ^a,b^ Different letters indicate significantly different values at *p* < 0.05.

## Supplementary Materials

The following are available online at https://www.mdpi.com/article/10.3390/toxins13040293/s1, Figure S1: Aggregation and sedimentation of microplastic particles in the cultures of *Alexandrium pacificum* ATHK during the later stage.
