# Supplementary material for: Effects of Polystyrene Microplastics on Growth and Toxin Production of Alexandrium pacificum
Source: Toxins (Basel). 2021 Apr 20;13(4):293. doi: 10.3390/toxins13040293 (PMC8074784; doi:10.3390/toxins13040293)
Supplement: Supplementary file 1 [file toxins-13-00293-s001.zip › toxins-1191345 - SI - corrected.pdf]

Supplementary Materials: Effects of Polystyrene Microplastics on Growth and Toxin Production of *Alexandrium pacificum*

Chao Liu, Jiangbing Qiu, Zhixuan Tang, Hong Hu, Fanping Meng and Aifeng Li

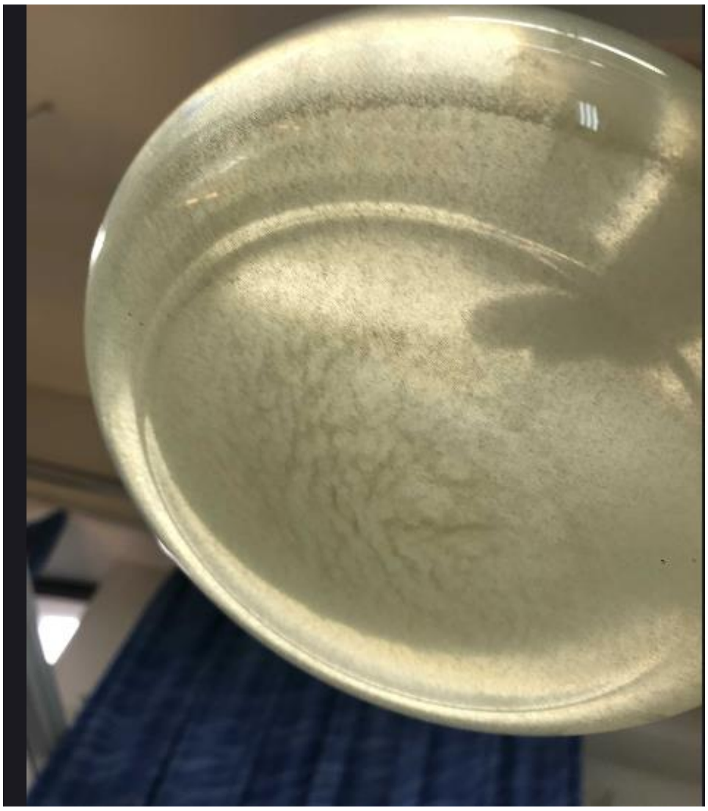

Figure S1. Aggregation and sedimentation of microplastic particles in the cultures of *Alexandrium pacificum* ATHK during the later stage.
